# Supplementary material for: The Presence of Two MyoD Genes in a Subset of Acanthopterygii Fish Is Associated with a Polyserine Insert in MyoD1
Source: J Dev Biol. 2023 Apr 28;11(2):19. doi: 10.3390/jdb11020019 (PMC10204381; doi:10.3390/jdb11020019)
Supplement: Supplementary file 1 [file jdb-11-00019-s001.zip › jdb-2330021-supplementary.pdf]

SUPPLEMENTAL MATERIAL

Table S1
Listed in this table are the sequences (5' to 3') for primers used for cloning amplification of cDNAs representing A.alcalica MyoD1 and MyoD2. Products were cloned into pGEM T-Easy to make antisense cRNA probes for in situ hybridisation analysis.

**Primer sets for generating probes**	
AaMyoD1 forward	5' CCCACCGCTGATGATTTCTAT 3'
AaMyoD1 reverse	5' GCTTCGTCTTCTGGTTGTCT 3'
AaMyoD2 forward	5' ATGGATCTGTCAGACTTTCCCTTCGTT 3'
AaMyoD2 reverse	5' ACAAATGTTGCTGGACTCGGCAGACAG 3'

Table S2
Listed in this table are the sequences (5' to 3') for primers used for cloning full length HA-tagged products that were directionally cloned into the EcoR1 and Xba1 sites in pCS2+ to generate synthetic mRNAs for expression in Xenopus.

**Primer sets for overexpression plasmids**	
AaMyoD1 forward	5' AGAGA**GGAATTC**ATGGAGTTGTCGGATATCTCTTTC 3'
AaMyoD1-HA reverse	5'AGAGAG**TCTAGA**AGCATAATCTGGAACATCATATGGATATAGGACTTGGTAAATCAGGTTGGGGTCCTGGCTGCC 3'
AaMyoD2 forward	5' AGAGAG**GAATTC**ATGGATCTGTCAGACTTTCCCTTCGTT 3'
AaMyoD2-HA reverse	5'AGAGAG**TCTAGA**AGCATAATCTGGAACATCATATGGATAACAAATGTTGCTGGACTCGGCAGACAG 3'
DrMyoD1 forward	5' AGAGA**GGAATTC**ATGGAGTTGTCGGATATCC 5'
DrMyoD1-HA reverse	5'AGAGAG**TCTAGA**AGCATAAATCTGGAACATCATATGGATAAAGCACTTGATAAATGGTTTCCTG 3'

Table S3
Listed in this table are the sequences (5' to 3') for primers used for qPCR to measure transcriptional activity of MyoD1 proteins in Xenopus explants.

**Primer sets for qPCR**	
X.laevis act3 forward	5' TCACAACAGCTGAAAGGGAGAT 3'
X.laevis act3 reverse	5' AAGTCCAGAGCCACATAGGC 3'
X.laevis myh4 forward	5' GTGCGTTGTTTGATTCCCAAT 3'
X.laevis myh4 reverse	5' GCTGGTGGATGAGGAGATGGT 3'
X.laevis dicer forward	5' GGCTTTTACACATGCCTCTTACC 3'
X.laevis dicer reverse	5' GTCCAAAATTGCATCTCCAAG 3'

SUPPLEMENTAL FIGURES

```
O.alcalica myoD1   MELS.....DISFPIP........TADDFYDDPCFNTSDMHFFEDLDPRLVHVG.LLKPD
     Danio myoD1   MELS.....DIPFPIP........SADDFYDDPCFNTNDMHFFEDLDPRLVHVS.LLKPD
     Mouse myoD1   MELLSPPLRDIDLTGPDGSLCSFETADDFYDDPCFDSPDLRFFEDLDPRLVHVGALLKPE

O.alcalica myoD1   DSSSSSSSSPSSSSSSPSSLLHLHHHAEVEDDEHVRAPSGHHQAGRCLLWACKACKRKTT
     Danio myoD1   E.....................HHHIE...DEHVRAPSGHHQAGRCLLWACKACKRKTT
     Mouse myoD1   E...HAHFST..........AVHPGPGAREDEHVRAPSGHHQAGRCLLWACKACKRKTT

O.alcalica myoD1   NADRRKAATLRERRRLSKVNDAFETLKRCTTANPNQRLPKVEILRNAISYIESLQALLRG
     Danio myoD1   NADRRKAATMRERRRLSKVNDAFETLKRCTSTNPNQRLPKVEILRNAISYIESLQALLRS
     Mouse myoD1   NADRRKAATMRERRRLSKVNEAFETLKRCTSSNPNQRLPKVEILRNAIRYIEGLQALLRD

O.alcalica myoD1   G.....QEDGFYP........VLEHYSGDSDASSPLSNCSDGMTDFNGPTCQTTRRRSYD
     Danio myoD1   ......QEDNYYP........VLEHYSGDSDASSPRSNCSDGMMDFMGPTCQTRRRNSYD
     Mouse myoD1   QDAAPPGAAAFYAPGPLPPGRGSEHYSGDSDASSPRSNCSDGMMDYSGPPSGPRRQNGYD

O.alcalica myoD1   SSSYFSETPNGGLKSERSSVVSSLDCLSSIVERISTDN..SSLLPPADGPG.SPTTTTTV
     Danio myoD1   SS.YFNDTPNADARNNKNSVVSSLDCLSSIVERISTETPACPVLSVPEGHEESPCSPHEG
     Mouse myoD1   TA.YYSEAVR.ESRPGKSAAVSSLDCLSSIVERISTDSPAAPALLLADAPPESPPGPPEG

O.alcalica myoD1   P..VGEAGTATATAQVSSPTG..SQDPNLIYQVLI
     Danio myoD1   S..VLSDTGTTAPSPTSCPQ...QQAQETIYQVL.
     Mouse myoD1   ASLSDTEQGTQTPSPDAAPQCPAGSNPNAIYQVL.
```

Supplemental Figure S1. Alignment of MyoD1 proteins from *Oreochromis alcalica, Danio rerio, and Mus musculus* showing highly conserved amino acid sequences in regions known to be essential for transcriptional activity (underlined): the transcriptional activation domain (TAD), Histidine/Cysteine (H/C) rich domain, and the bHLH domain. Residues implicated in protein stability are also conserved across the MyoD1 proteins and highlighted: putative phosphorylation targets of proline directed kinases (SP); Lysine (K) substrates for ubiquitin ligase; Tyrosines (Y) that can be targets of the kinase MEK1. Two additional sites for proline directed phosphorylation are found in the poly serine domain in *O.alcalica* MyoD1.

```
Amazon_molly_Poecilia_formosa               PRLVHVVPLKPEDSCSPSSSS.......SSSSPSPCS..HLHHQLQH..
Ballan_wrasse_Labrus_bergylta               PRLVHVGLLKPDDSSSSPSPSP.....SSCSSSSPSSLLHLHHH.....
Barramundi_perch_Lates_calcarifer           PRLVHVGLLKPDDSSSSSSSSP.....SSSSSSPSSLLHLHHHH....
Bicolor_damselfish_Stegastes_partitus       PRLVHVGLLKPDDSSSSSSSSP.....SSSSSSPSSLLHLHHH.....
Bluntsnouted_clingfish_Gouania_willdeno     PRLVHAGLH..ESSSLSSSSSP.SSSSSSSSSSPSSSLLHHHHHHHHH
Burtons_mouthbrooder_Haplochromis_burto     PRLVHVGLLKPDDSSSSSSSSP......SSSSSPSSLLHLHHH.....
Channel_bull_blenny_Cottoperca_gobio        PRLVHVGLLKPDDSSSSPSPSP.....SSSSSSPSSLLHLHHH.....
Climbing_perch_Anabas_testudineus           PRLVHVGLLKPDDSSSSSSSSP.......SSSSSPSSLLHLHHN.....
Clown_anemonefish_Amphiprion_ocellaris      PRLVHVGLLKPDDSSSSSSPSP....SSSSSSSPSSLLHLHHH.....
Eastern_happy_Astatotilapia_calliptera      PRLVHVGLLKPDDSSSSSSSSP......SSSSSPSSLLHLHHH.....
Greater_amberjack_Seriola_dumerili          PRLVHVGLLKPDDSSSSTSSSP.....SSSSSSPSSLLHLHHH.....
Indian_glassy_fish_Parambassis_ranga        PRLVHVGLLKPDDSSSSSSSSP.....SSSSASPSSLLHLHHHHHH..
Indian_medaka_Oryzias_melastigma            PRLVHVGLLKPDDSSSSSSSSP......CSSSSPSSILHLHQR.....
Japanese_medaka_Oryzias_latipes             PRLVHGGLLKPDDSSSSPSSSP......CSSSSPSSVLHVHQR.....
Large_yellow_croaker_Larimichthys_croce     PRLVHVGLLKPDDSSSSPSPSP....SSSSASSSPSSLLHLHHH.....
Lyretail_cichlid_Neolamprologus_brichar     PRLVHVGLLKPDDSSSSSSSSP......SSSSSPSSLLHLHHH.....
Makobe_Island_cichlid_Pundamilia_nyerer     PRLVHVGLLKPDDSSSSSSSSP......SSSSSPSSLLHLHHH.....
Midas_cichlid_Amphilophus_citrinellus       PRLVHVGLLKPDDSSSSSSCSP......SSSSSPSSLLHLNHH.....
Mummichog_Fundulus_heteroclitus             PRLVHVVPMKPEDSSSPSSS........LSSSSLSPSS..HLHHHLQH..
Ocean_sunfish_Mola_mola                     PRLVHVGLLKPDDTSSSASSSSSSPSPSSSTSSSPSSLLHLHHR.....
Orange_clownfish_Amphiprion_percula         PRLVHVGLLKPDDSSSSSSPSP......SSSSSPSSLLHLHHH.....
Oreochromis_Alcolapia_alcalica              PRLVHVGLLKPDDSSSSSSSSP......SSSSSPSSLLHLHHH.....
Platyfish_Xiphophorus_maculatus             PRLVHGVPLKPEDSFSPSSS........LSSSSPSPCS..HLHHQLQH..
Sheepshead_minnow_Cyprinodon_variegatus     PRLVHVVPLKPEDSSSSLSS.........SSSSSPYS..HLHHHLQR..
Shortfin_molly_Poecilia_mexicana            PRLVHVVPLKPEDSCSPSSSS........SSSSPSPCS..HLHHQLQH..
Siamese_fighting_fish_Betta_splendens       PRLVHVGLLKPDDSSSSSSSSP......SSSSSPSSLLHLHHN.....
Swamp_eel_Monopterus_albus                  PRLVHVGLLKPDDSSSSTASSP.....SSTSSSPSSLLHLHHH.....
Tetraodon_Tetraodon_nigroviridis            PRLVHTLLKPDDCCSSSSLSP.......SSSSSPSSLQHHHHH....
Tilapia_Oreochromis_niloticus               PRLVHVGLLKPDDSSSSSSSSP......SSSSSPSSLLHLHHH.....
Tongue_sole_Cynoglossus_semilaevis          PRLVHVGLLKPDDSSSSSSPS.......SSSSSPSSLLHLHHH.....
Western_mosquitofish_Gambusia_affinis       PRLVHGVPLKPEDSCSPSSSSS.....LSSSSPSPCP..HLHHQLQH..
Zebra_mbuna_Maylandia_zebra                 PRLVHVGLLKPDDSSSSSSSSP......SSSSSPSSLLHLHHH.....
```

Supplemental Figure S2. Alignment of polyserine insert regions from multiple teleost MyoD1 proteins. The grey region is conserved in all vertebrate MyoD1 sequences as part of the N-terminal activation domain. The Histidine highlighted in green indicates the beginning of the H/C rich region, where conservation with all vertebrate MyoD1 proteins is re-established. Potential sites for proline directed kinase activity are indicated in yellow.

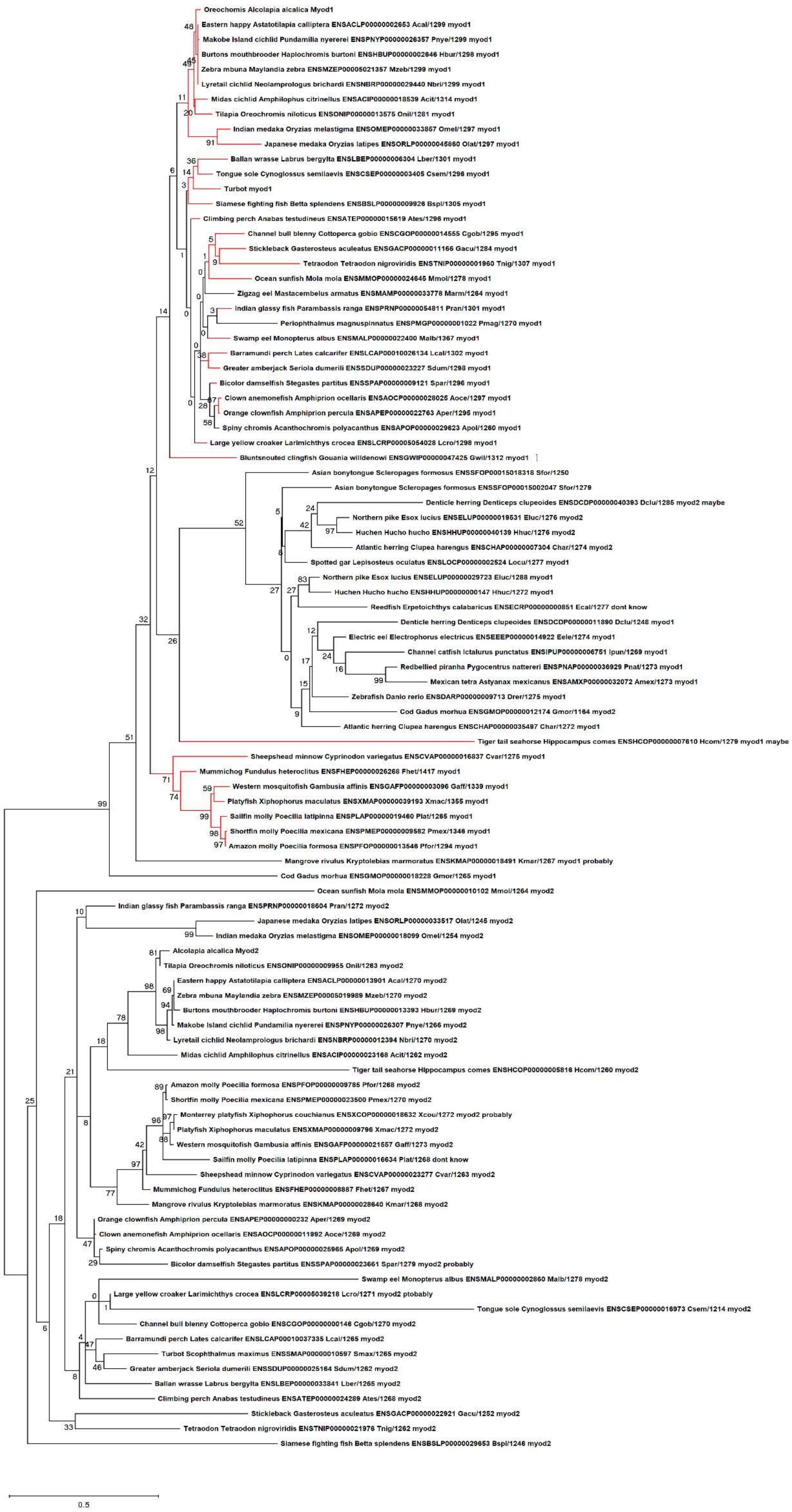
Oreochomis Alcolapia alcalica Myod1
Eastern happy Astatotilapia calliptera ENSACLP00000002653 Acal/1299 myod1
Makobe Island cichlid Pundamilia nyererei ENSPNYP00000026357 Pnye/1299 myod1
Burtons mouthbrooder Haplochromis burtoni ENSHBUP00000002646 Hbur/1298 myod1
Zebra mbuna Maylandia zebra ENSMZEP00005021357 Mzeb/1299 myod1
Lyretail cichlid Neolamprologus brichardi ENSNBRP00000029440 Nbri/1299 myod1
Midas cichlid Amphilophus citrinellus ENSACIP00000018539 Acit/1314 myod1
Tilapia Oreochromis niloticus ENSONIP00000013575 Onil/1281 myod1
Indian medaka Oryzias melastigma ENSOMEP00000033857 Omel/1297 myod1
Japanese medaka Oryzias latipes ENSORLP00000045860 Olat/1297 myod1
Ballan wrasse Labrus bergylta ENSLBEP00000006304 Lber/1301 myod1
Tongue sole Cynoglossus semilaevis ENSCSEP00000003405 Csem/1296 myod1
Turbot myod1
Siamese fighting fish Betta splendens ENSBSLP00000009926 Bspl/1305 myod1
Climbing perch Anabas testudineus ENSATEP00000015619 Ates/1296 myod1
Channel bull blenny Cottoperca gobio ENSCGOP00000014555 Cgob/1295 myod1
Stickleback Gasterosteus aculeatus ENSGACP00000011166 Gacu/1284 myod1
Tetraodon Tetraodon nigroviridis ENSTNIP00000001960 Tnig/1307 myod1
Ocean sunfish Mola mola ENSMMOP00000024645 Mmol/1278 myod1
Zigzag eel Mastacembelus armatus ENSMAMP00000033778 Marm/1264 myod1
Indian glassy fish Parambassis ranga ENSPRNP00000054811 Pran/1301 myod1
Periophthalmus magnuspinnatus ENSPMGP00000001022 Pmag/1270 myod1
Swamp eel Monopterus albus ENSMALP00000022400 Malb/1367 myod1
Barramundi perch Lates calcarifer ENSLCAP00010026134 Lcal/1302 myod1
Greater amberjack Seriola dumerili ENSSDUP00000023227 Sdum/1298 myod1
Bicolor damselfish Stegastes partitus ENSSPAP00000009121 Spar/1296 myod1
Clown anemonefish Amphiprion ocellaris ENSAOCP00000028025 Aoce/1297 myod1
Orange clownfish Amphiprion percula ENSAPEP00000022763 Aper/1295 myod1
Spiny chromis Acanthochromis polyacanthus ENSAPOP00000029623 Apol/1260 myod1
Large yellow croaker Larimichthys crocea ENSLCRP00005054028 Lcro/1298 myod1
Bluntsnouted clingfish Gouania willdenowi ENSGWIP00000047425 Gwil/1312 myod1
Asian bonytongue Scleropages formosus ENSSFOP00015018318 Sfor/1250
Asian bonytongue Scleropages formosus ENSSFOP00015002047 Sfor/1279
Denticle herring Denticeps clupeoides ENSDCDP00000040393 Dclu/1285 myod2 maybe
Northern pike Esox lucius ENSELUP00000019531 Eluc/1276 myod2
Huchen Hucho hucho ENSHHUP00000040139 Hhuc/1276 myod2
Atlantic herring Clupea harengus ENSCHAP00000007304 Char/1274 myod2
Spotted gar Lepisosteus oculatus ENSLOCP00000002524 Locu/1277 myod1
Northern pike Esox lucius ENSELUP00000029723 Eluc/1288 myod1
Huchen Hucho hucho ENSHHUP00000000147 Hhuc/1272 myod1
Reedfish Erpetoichthys calabaricus ENSECRP00000000851 Ecal/1277 dont know
Denticle herring Denticeps clupeoides ENSDCDP00000011890 Dclu/1248 myod1
Electric eel Electrophorus electricus ENSEEEP00000014922 Eele/1274 myod1
Channel catfish Ictalurus punctatus ENSIPUP00000006751 Ipun/1269 myod1
Redbellied piranha Pygocentrus nattereri ENSPNAP00000036929 Pnat/1273 myod1
Mexican tetra Astyanax mexicanus ENSAMXP00000032072 Amex/1273 myod1
Zebrafish Danio rerio ENSDARP00000009713 Drer/1275 myod1
Cod Gadus morhua ENSGMOP00000012174 Gmor/1164 myod2
Atlantic herring Clupea harengus ENSCHAP00000035497 Char/1272 myod1
Tiger tail seahorse Hippocampus comes ENSHCOP00000007610 Hcom/1279 myod1 maybe
Sheepshead minnow Cyprinodon variegatus ENSCVAP00000016837 Cvar/1275 myod1
Mummichog Fundulus heteroclitus ENSFHEP00000026268 Fhet/1417 myod1
Western mosquitofish Gambusia affinis ENSGAFP00000003096 Gaff/1339 myod1
Platyfish Xiphophorus maculatus ENSXMAP00000039193 Xmac/1355 myod1
Sailfin molly Poecilia latipinna ENSPLAP00000019460 Plat/1265 myod1
Shortfin molly Poecilia mexicana ENSPMEP00000009582 Pmex/1346 myod1
Amazon molly Poecilia formosa ENSPFOP00000013546 Pfor/1294 myod1
Mangrove rivulus Kryptolebias marmoratus ENSKMAP00000018491 Kmar/1267 myod1 probably
Cod Gadus morhua ENSGMOP00000018228 Gmor/1265 myod1
Ocean sunfish Mola mola ENSMMOP00000010102 Mmol/1264 myod2
Indian glassy fish Parambassis ranga ENSPRNP00000018604 Pran/1272 myod2
Japanese medaka Oryzias latipes ENSORLP00000033517 Olat/1245 myod2
Indian medaka Oryzias melastigma ENSOMEP00000018099 Omel/1254 myod2
Alcolapia alcalica Myod2
Tilapia Oreochromis niloticus ENSONIP00000009955 Onil/1263 myod2
Eastern happy Astatotilapia calliptera ENSACLP00000013901 Acal/1270 myod2
Zebra mbuna Maylandia zebra ENSMZEP00005019989 Mzeb/1270 myod2
Burtons mouthbrooder Haplochromis burtoni ENSHBUP00000013393 Hbur/1269 myod2
Makobe Island cichlid Pundamilia nyererei ENSPNYP00000026307 Pnye/1266 myod2
Lyretail cichlid Neolamprologus brichardi ENSNBRP00000012394 Nbri/1270 myod2
Midas cichlid Amphilophus citrinellus ENSACIP00000023168 Acit/1262 myod2
Tiger tail seahorse Hippocampus comes ENSHCOP00000005816 Hcom/1260 myod2
Amazon molly Poecilia formosa ENSPFOP00000009785 Pfor/1268 myod2
Shortfin molly Poecilia mexicana ENSPMEP00000023500 Pmex/1270 myod2
Monterrey platyfish Xiphophorus couchianus ENSXCOP00000018632 Xcou/1272 myod2 probably
Platyfish Xiphophorus maculatus ENSXMAP00000009796 Xmac/1272 myod2
Western mosquitofish Gambusia affinis ENSGAFP00000021557 Gaff/1273 myod2
Sailfin molly Poecilia latipinna ENSPLAP00000016634 Plat/1268 dont know
Sheepshead minnow Cyprinodon variegatus ENSCVAP00000023277 Cvar/1263 myod2
Mummichog Fundulus heteroclitus ENSFHEP00000008887 Fhet/1267 myod2
Mangrove rivulus Kryptolebias marmoratus ENSKMAP00000028640 Kmar/1268 myod2
Orange clownfish Amphiprion percula ENSAPEP00000000232 Aper/1269 myod2
Clown anemonefish Amphiprion ocellaris ENSAOCP00000011992 Aoce/1269 myod2
Spiny chromis Acanthochromis polyacanthus ENSAPOP00000025965 Apol/1269 myod2
Bicolor damselfish Stegastes partitus ENSSPAP00000023661 Spar/1279 myod2 probably
Swamp eel Monopterus albus ENSMALP00000002860 Malb/1278 myod2
Large yellow croaker Larimichthys crocea ENSLCRP00005039218 Lcro/1271 myod2 ptobably
Tongue sole Cynoglossus semilaevis ENSCSEP00000016973 Csem/1214 myod2
Channel bull blenny Cottoperca gobio ENSCGOP00000000146 Cgob/1270 myod2
Barramundi perch Lates calcarifer ENSLCAP00010037335 Lcal/1265 myod2
Turbot Scophthalmus maximus ENSSMAP00000010597 Smax/1265 myod2
Greater amberjack Seriola dumerili ENSSDUP00000025164 Sdum/1262 myod2
Ballan wrasse Labrus bergylta ENSLBEP00000033841 Lber/1265 myod2
Climbing perch Anabas testudineus ENSATEP00000024289 Ates/1268 myod2
Stickleback Gasterosteus aculeatus ENSGACP00000022921 Gacu/1252 myod2
Tetraodon Tetraodon nigroviridis ENSTNIP00000021976 Tnig/1262 myod2
Siamese fighting fish Betta splendens ENSBSLP00000029653 Bspl/1246 myod2
0.5

Supplemental Figure S3 (previous page). Phylogenetic tree derived from the analysis of the amino acid sequences of 97 teleost MyoD proteins. MyoD1 and MyoD2 proteins were analysed using MEGAX using the JTT w/freq method and gamma distribution. 59 MyoD1 [36 with a poly-serine insertion] and 38 MyoD2 from 54 teleost species clade separately; where MyoD1 containing and not containing a poly-serine insertion clade as MyoD1. Analysis conducted on clades is supported by a bootstrap value of 99, scale bar represents a genetic distance of 0.5 amino acid substitutions per site. Those MyoD1 with a poly serine insertion are indicated by red branches red.

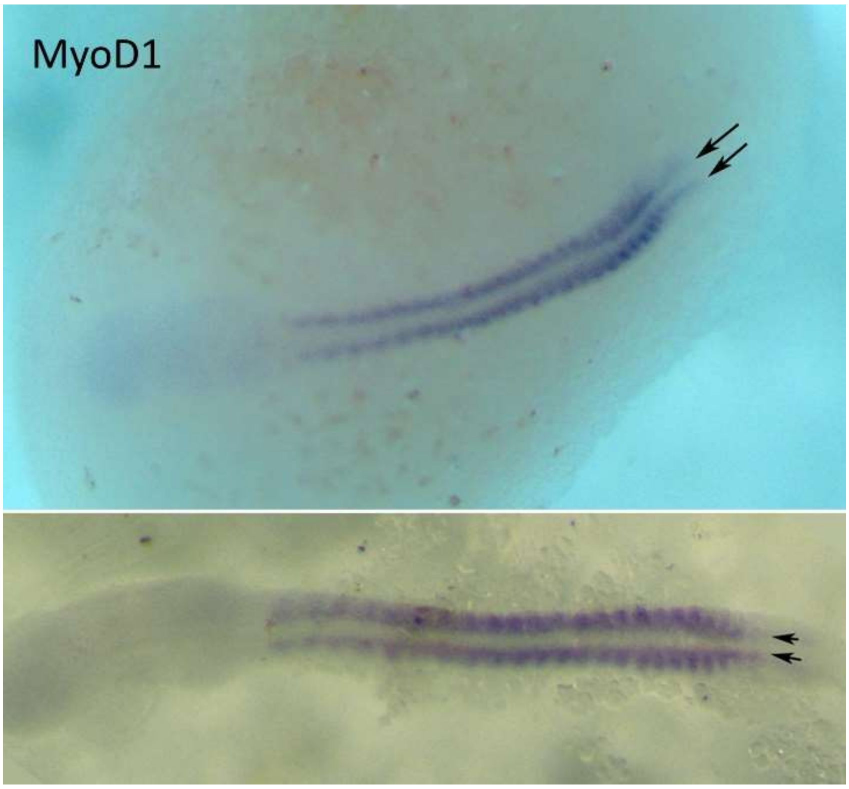

Supplemental Figure S4. The expression of *MyoD1* in pre-hatch stage *Oreochromis (Alcolapia) alcalica* embryos. The top panel shows a whole mount in situ hybridisation as a dorsal view, with anterior to the left. The bottom panel is a similar stage embryo as a flat-mount, where the yolk has been removed so the embryo could be flattened to improve visualisation of the posterior expression. The arrows in both panels indicate *MyoD1* expression in the putative adaxial region.

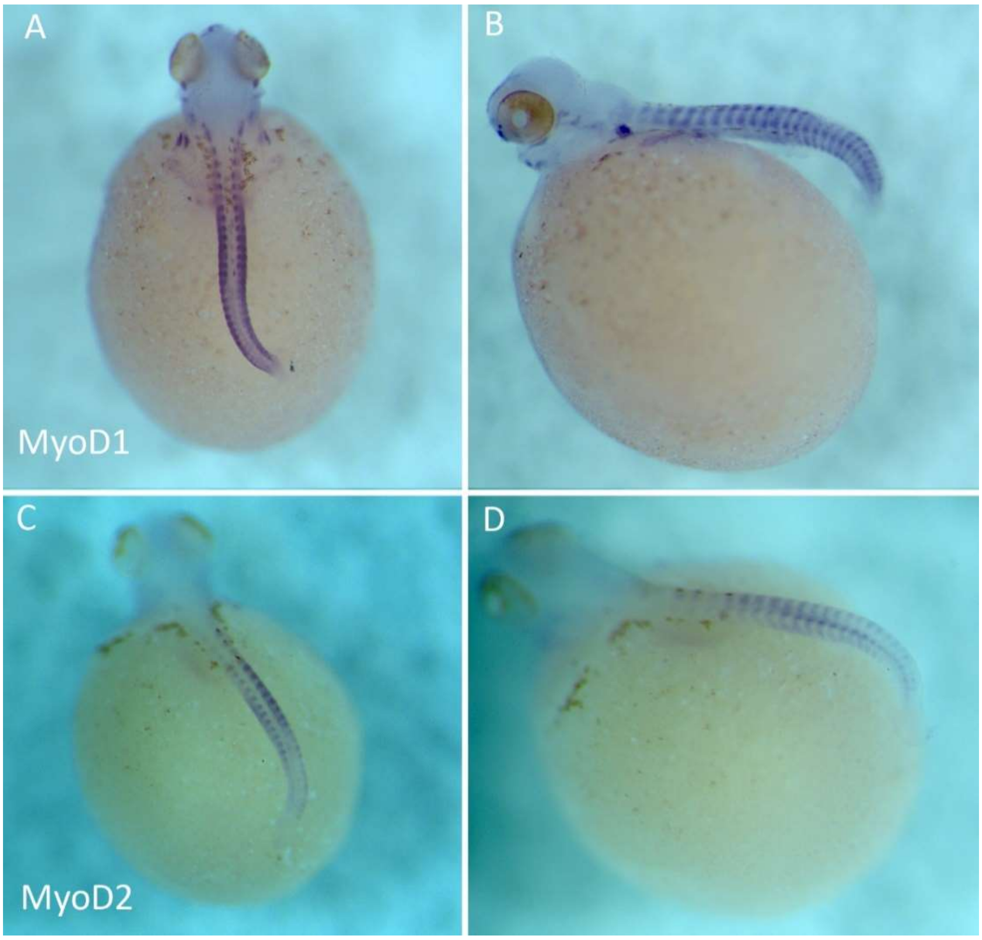

Supplemental Figure S5. Expression of MyoD1 (A,B) and MyoD2 (C,D) in stage *Oreochromis (Alcolapia) alcalica* embryos at approximately 3 days post fertilisation. A,C are dorsal views; B,D are lateral views with anterior to the left.
